# Valorization of Greenhouse Horticulture Waste from a Biorefinery Perspective

**DOI:** 10.3390/foods10040814

**Published:** 2021-04-09

**Authors:** Antonio D. Moreno, Aleta Duque, Alberto González, Ignacio Ballesteros, María José Negro

**Affiliations:** Advanced Biofuels and Bioproducts Unit, Department of Energy, Research Centre for Energy, Environment and Technology (CIEMAT), Avda. Complutense 40, 28040 Madrid, Spain; david.moreno@ciemat.es (A.D.M.); aleta.duque@ciemat.es (A.D.); alb.gonzalez@ciemat.es (A.G.); ignacio.ballesteros@ciemat.es (I.B.)

**Keywords:** tomato waste, enzymatic hydrolysis, extrusion, sugar platform

## Abstract

Greenhouse cultivation and harvesting generate considerable amounts of organic waste, including vegetal waste from plants and discarded products. This study evaluated the residues derived from tomato cultivation practices in Almería (Spain) as sugar-rich raw materials for biorefineries. First, lignocellulose-based residues were subjected to an alkali-catalyzed extrusion process in a twin-screw extruder (100 °C and 6–12% (*w/w*) NaOH) to assess maximum sugar recovery during the subsequent enzymatic hydrolysis step. A high saccharification yield was reached when using an alkali concentration of 12% (*w*/*w*), releasing up to 81% of the initial glucan. Second, the discarded tomato residue was crushed and centrifuged to collect both the juice and the pulp fractions. The juice contained 39.4 g of sugars per 100 g of dry culled tomato, while the pulp yielded an extra 9.1 g of sugars per 100 g of dry culled tomato after an enzymatic hydrolysis process. The results presented herein show the potential of using horticulture waste as an attractive sugar source for biorefineries, including lignocellulose-based residues when effective fractionation processes, such as reactive extrusion technology, are available.

## 1. Introduction

Biorefineries are industries capable of converting a vast variety of biomass feedstocks (e.g., lignocellulose, algal biomass, the organic fraction of municipal solid wastes, industrial wastes) into bio-based renewable products, including biofuels, nutraceuticals, bioplastics, fertilizers, and/or other chemicals [1]. Food-related waste is considered among the most attractive raw materials for biorefineries due to its low costs and its high organic matter content and the presence of interesting and potentially valuable components (e.g., proteins, sugars, oils, and phenolic compounds) [2,3,4]. In addition, the use of food waste as biorefinery feedstock represents an alternative to conventional waste management methods and contributes to reducing the amount of discarded residues, offering the opportunity to improve the economic and environmental performance of the food sector.

In Europe, the fruit and vegetable sector reached a total annual output value of 57 billion euros in 2018 [5]. Among the market products, tomatoes were by far the main vegetable product, with 16.6 million tons and occupying 10% of the total vegetable cultivated area, with Spain and Italy as major producers (60% of the total produced volume) [6,7]. After harvesting, this crop generates annually over 3 million metric tons of waste [8], mainly including plant waste (leaves and branches generated from pruning and maintenance operation, as well as the main stem, branches, and leaves collected at the end of the growing cycle) and the discarded tomatoes that do not meet the required quality standards for sale. In the particular case of Almería, Spain, where more than 75% of the country’s total greenhouse production occurs, the amount of tomato plant waste comes to around 470,000 tons/year, considering both data from the area for growing tomato in greenhouse crop and the estimated value of waste generation (49 t tomato plant waste per ha) [9]. On the other hand, the number of discarded fruits greatly varies from one year to another and may represent up to 25% of the total crop waste [2,10,11].

Vegetal waste is characterized by high moisture and salt content and is highly biodegradable [12]. These residues usually end up in landfills or are used as raw material for compost production [13]. They can also be transferred to an external waste management company or an authorized recycling plant for their treatment [10], which creates an extra cost to the farms. A small proportion, especially discarded fruits, can be used for livestock feeding as well. In contrast, the use of these waste products as raw material for the production of biofuels and/or bio-based products in biorefinery plants is nowadays seen as an interesting scenario for the sustainable valorization of these residues [14].

The main components of vegetal waste include lipids, nonpolar compounds, structural (e.g., cellulose, hemicellulose) and nonstructural carbohydrates (e.g., sucrose), proteins, and lignin [15]. Among them, carbohydrates have high potential for being used in a wide range of fermentation-based processes within the sugar platform biorefinery. Here, microbial oil production constitutes a research area with growing interest, as they can be widely employed in functional food formulations or considered as a sustainable alternative to fossil fuels. Currently, major challenges for microbial oil production include the development of multiproduct biorefineries and the use of low-cost feedstocks to establish an economically feasible and sustainable process [16]. In this context, food-derived waste may play an important role as feedstock for microbial oil production due to its low cost, high carbohydrate content, and potential as a source of secondary products such as bioactive compounds that can contribute to the process economy [4].

Carbohydrates from discarded vegetable products can be easily extracted by collecting the corresponding juice after a crushing procedure. In contrast, the lignocellulosic nature of vegetal plant waste makes necessary a pretreatment process for effective fractionation of the biomass. Several pretreatment technologies have been developed for lignocellulosic feedstocks, including physical, chemical, physicochemical, and biological pretreatments. Extensive research efforts have been made on identifying, evaluating, developing, and demonstrating appropriate pretreatment technologies to increase the subsequent saccharification process with low enzyme dosages and shorter conversion times [17].

Extrusion technology is a pretreatment method based on mechanical deconstruction of the biomass matrix through the shear forces produced by a screw spinning into a tight, controlled-temperature barrel. During extrusion, the materials are subjected to heating, mixing, and shearing stresses inside the extruder barrel, resulting in chemical and physical changes. Extrusion has been proposed as a versatile pretreatment with promising features for increasing enzymatic hydrolysis of lignocellulose [18] and as a step before anaerobic digestion for methane production [19]. This pretreatment allows different process configurations and can therefore be adapted to the addition of chemicals as alkalis [20], acids [21], ionic liquids [22], or deep eutectic solvents [23] and/or biological catalysts (enzymes) [24,25]. In contrast to other pretreatment methods such as thermochemical processes, extrusion pretreatment does not always require harsh conditions (i.e., high pressures and/or temperatures). This significantly reduces the concentration of biomass degradation products generated during the pretreatment step, thus reducing the inhibitory capacity of the resulting pretreated biomass. In addition, extrusion allows working at a high biomass concentration and in a continuous operation mode, which is crucial during the scaling up of the process. Extrusion has successfully been used as a pretreatment to increase the enzymatic hydrolysis yields of different herbaceous biomass, such as sugarcane bagasse [26], barley straw [27], corn stover [28], and *Miscanthus* [29]. Similarly, woody biomass including *Eucalyptus* [30] or olive tree pruning [31] has recently been pretreated by extrusion, showing promising results.

The present work aims at assessing the suitability of using the resulting vegetal waste derived from tomato cultivation practices as a sugar source for biorefinery-based processes. In order to maximize sugar recovery, the vegetal tomato plant waste (VTPW) was pretreated by reactive extrusion with alkali at different concentrations, while discarded tomatoes were crushed and centrifuged to separate the juice from the pulp. These fractionation procedures were evaluated in terms of sugar recovery yields after a subsequent saccharification process of each pretreated fraction, when necessary. The results highlight the great potential of vegetal waste for the production of carbohydrates, which can be further converted into a wide range of bio-based processes such as microbial oil production.

## 2. Materials and Methods

### 2.1. Raw Materials

Culled tomato (CT, damaged products during harvesting and products not suitable as food, such as products with mold) and VTPW were kindly supplied by Albaida Residuos S.L. (Almería, Spain). These residues had 95.0% and 45.7% moisture content, respectively. Both CT and VTPW were subjected to different biomass fractionation process for sugar recovery according to Section 2.2 and Section 2.4. In the particular case of VTPW, before further processing, the biomass was dried at 40 °C until 10% moisture and milled to 4 mm particle size using a Retsch ZM200.

A small fraction of each raw material was taken for biomass characterization according to Section 2.6.

### 2.2. Extrusion Pretreatment

VTPW was subjected to extrusion pretreatment for increasing the accessibility of structural carbohydrates. Extrusion pretreatment was carried out using a co-rotating twin-screw extruder (Clextral Processing Platform Evolum^®^ 25 A110, Clextral, France). The twin-screw extruder consisted of six modules of 100 mm, each equipped with temperature control, and a twin-screw with a length-to-diameter ratio L/D of 24:1.

**Figure 1:**
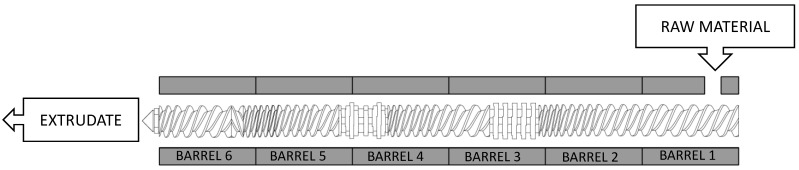
Screw configuration for the extrusion of vegetal tomato plant waste (VTPW) in a twin-screw extruder.

Operating conditions were set to achieve moderate values of NaOH/dry matter ratio, 6 and 12% (*w/w*), 100 °C barrel temperature, and 150 rpm rotation speed. The screw profile configuration and operation conditions were chosen based on previous experience of the CIEMAT team on extrusion pretreatment [30]. Prior to the extrusion process, VTPW biomass was thoroughly mixed with the alkali solution for 20 min and then fed into the extruder by hand. After the first run, a portion sample of extruded biomass or extrudate (denoted as one-run) was separated for composition analysis and enzymatic hydrolysis test, while the rest was extruded again. The operation was repeated twice, and the resulting pretreated material was denoted as three-run. Figure 1 illustrates the diagram of the screw profile configuration.

### 2.3. Extrudate Characterization

After extrusion, 100 g of extrudate was water-washed and filtered to remove any residual alkali, following the procedure described by the National Renewable Energy Laboratory (NREL) to determine water-insoluble solids (WIS) content [32]. The resulting WIS fraction were analyzed for cellulose, hemicellulose (considered as sum of xylan, galactan, arabinan, and mannan), lignin, ash, and acetyl-groups content after two-step sulfuric acid hydrolysis [33], as explained in Section 2.6.

### 2.4. Culled Tomatoes Fractionation

Two kilograms of culled tomatoes were subjected to a crushing process followed by a centrifugation step to separate the juice and the pulp. After centrifugation, the resulting juice, or liquid fraction (LF-CT), was analyzed by high performance liquid chromatography (HPLC) to determine its sugars content, while the pulp, or solid fraction (SF-CT), was freeze-dried and analyzed for cellulose, hemicellulose, acid-insoluble solids, ash, and nitrogen content.

### 2.5. Enzymatic Hydrolysis Test

The WIS obtained after extrusion of VTPW was enzymatically hydrolyzed using a commercial enzyme blend (Cellic Ctec2) purchased from SIGMA (Ref. SAE0020). This preparation contains cellulases, hemicellulases, and β-glucosidase enzymes. Enzymatic hydrolysis assays were performed in triplicate, using 100-mL Erlenmeyer flasks. Assays were run in 50 mM sodium citrate buffer (pH 5) at 5 and 10% (*w*/*v*) substrate concentration (dry weight, DW), 50 °C, 150 rpm, and enzyme loading of 15 FPU/g DW of substrate. Representative samples were taken after 24, 48, and 72 h and centrifuged at 9300× *g* for 10 min. Supernatants were analyzed by HPLC to determine the sugars concentration.

Enzymatic hydrolysis yields (EH) were estimated considering the glucose/xylose produced during enzymatic hydrolysis (after subtracting the sugar content from the enzyme preparation), which is referred to the potential glucose/xylose (calculated based on the glucan/xylan content in the WIS) and was expressed as a percentage (denoted EH_G_ and EH_X_). Average values ± SD of the three replicates were presented.

SF-CT was also subjected to enzymatic hydrolysis in 100-mL Erlenmeyer flasks at 10% (*w*/*v*) substrate loadings, 50 °C, and 150 rpm for 48 h. With the aim of maximizing enzymatic hydrolysis yields from SF-CT, different enzyme preparations with different hydrolytic activities were used as follows: (1) 1% (*w/w*) N22086/g DW of substrate; (2) 0.1% NS22119/g DW of substrate; (3) 15 FPU Cellic CTec2/g DW of substrate. NS22086 and NS22119 were both provided by Novozymes (Denmark) and are enzymatic preparations with cellulases, xylanases, and carbohydrases (arabinase, β-glucanase, cellulase, hemicellulase, pectinase, and xylanase) activities, respectively.

### 2.6. Analytical Methods

Compositional analyses of raw biomass and pretreated solids (the WIS collected after extrusion of VTPW and the SF-CT) were determined according to the two-step acid hydrolysis protocol from the analytical methods for biomass described by NREL [33].

Sugar content (i.e., glucose, xylose, arabinose, mannose, galactose, fructose, and saccharose) was quantified by HPLC using a Waters 2695 liquid-chromatograph equipped with a refractive index detector. A CARBOSep CHO-682 LEAD column (Transgenomic, Omaha, NE, USA) operating at 75 °C with Milli-Q water (Millipore, Burlington, MA, USA) as mobile phase (0.5 mL/min) was employed for sugar separation.

Acetic acid was also quantified by HPLC (Waters, Milford, MA, USA) using a 410 Water refractive index detector and an Aminex HPX-87H (Bio-Rad Labs, Hercules, CA, USA) column operating at 65 °C with 5 mM H_2_SO_4_ (0.6 mL/min) as mobile phase.

### 2.7. Statistical Analysis

Statistical analysis was performed using Statgraphic Centurion XVII.I-X64 for Windows. Analysis of variance (ANOVA) was used for comparisons between assays at the 95% level. The Bonferroni’s post-test was used when appropriate.

## 3. Results and Discussion

### 3.1. Chemical Composition of Vegetal Tomato Plant Waste

Table 1 reports the chemical composition analysis of VTPW. The main component was extracts, accounting for 40.6% of the total DW. More than 85% of total extracted components were collected in aqueous media (35.0 ± 0.3%), and about 15% (5.6 ± 0.3%) corresponded to organic solvent (ethanol) extracts. Water-soluble materials may include inorganic compounds, nonstructural sugars, nitrogenous compounds, and other compounds, while ethanol-soluble components include chlorophyll, waxes, and other minor compounds. The main water-soluble components were carbohydrates, with 4.7% of the total aqueous extracts, glucose-based oligomers being the principal sugars (2.3%). Other nondetermined extracts include flavonoids, tannins, and terpenoids, among others [34]. The proportion of the extractive fraction found in the present study is higher than the values reported in the scientific literature. Extracts contents of 22%, 36%, and 27% have been reported in the aqueous phase from the post-harvested tomato plant, stems, and leaves, respectively [34,35]. These differences can be explained by the influence of several factors such as cultivation and climatic conditions, as well as the methodology used to determine extractives.

The second main component after extracts in VTPW was ash, showing a total content of 17.5% DW. Similar ash values have previously been reported for either leaves, stems, or whole tomato plants [15,36]. Nisticò et al. [35] reported ash concentration of up to 22% in this vegetal residue, and Gary et al. [37] estimated ash content of 0.12 g/g DW in leaves and 0.26 g/g DW in the corresponding stems, respectively. This high ash content may be associated with high mineral content in tomato plant tissues, which is typical of greenhouse crops in general. Furthermore, it is important to remark that about 50% of those inorganic compounds were extractable, and ash content decreased to 15.2% in the corresponding extract-free biomass (data not shown).

VTPW accounted for a total carbohydrate content of 20.6%, including total glucans, hemicelluloses, and water-soluble sugars. In addition, VTPW had a lignin content of up to 13.0% when considering both acid-insoluble (9.4%) and acid-soluble (3.6%) lignin. Although similar values have been found in the literature regarding lignin content of this residue [36], the carbohydrate content was lower compared to other reported values. For instance, Nisticò et al. [35] reported a total carbohydrate content (cellulose and hemicellulose) of up to 43.6% in post-harvest tomato plant. The difference could be due not only to the different analytical methods used, but also to the different origin of the residues; in our case, VTPW came from a waste management company, and the feedstock reported by Nisticò et al. [35] was obtained directly from experimental crops.

### 3.2. Extrusion Pretreatment of Vegetal Tomato Plant Waste

With the aim of evaluating the potential of VTPW as a sugar source, this biomass waste was first subjected to an alkali-catalyzed extrusion pretreatment to ease the accessibility of structural carbohydrates during the subsequent enzymatic hydrolysis step. Table 2 and Table 3 list the WIS content in the resulting extrudates, the corresponding mass yields (expressed as grams WIS/100 g raw material), and the complete chemical composition of the collected WIS fraction.

Independently of the number of runs during the extrusion process (one-run or three-run pretreated substrates), a clear correlation was observed between the NaOH concentration and the WIS content in the extrudates (*p* < 0.05). In this sense, the higher the NaOH concentration, the lower the WIS content in the extrudates. WIS content decreased from 56.6–56.9% to 46.8–49.5% after increasing the alkali concentration from 6 to 12% (*w/w*) (Table 2). Similarly, the recovery of insoluble solids in terms of mass yields also decreased from 58.3–58.7% to 54.4–51.1% when the amount of catalyst rose to 12%.

The compositional analysis of all collected WIS fractions at different runs and NaOH concentrations revealed important changes when compared to the non-pretreated VTPW. The alkali-catalyzed extrusion and subsequent washing generated a solid fraction with a higher content of carbohydrates (26.2–32.4%) and lignin (23.0–26.0%). This effect can be attributed to the solubilization of the extractable compounds that are present in the raw material, which significantly reduced the content of this fraction. Reaching a high carbohydrate concentration in the pretreated material is essential to yielding higher sugar concentrations during the subsequent saccharification process. These results therefore support the use of extrusion pretreatment for the fractionation of collected vegetal wastes from greenhouse crops.

In contrast to the results obtained in this study, Domenech et al. [38] reported no changes in cellulose content after subjecting olive stone to alkali extrusion, independently of pretreatment temperature or the concentration of soda. However, these authors observed a slight decrease in hemicellulose content of the collected WIS fraction after extrusion pretreatment. It should be noted that olive stone showed a lower extracts content (6.8%) when compared to that of VTPW biomass, which can explain the differences in terms of compositional analysis even when feedstocks are subjected to similar pretreatment processes.

Alkaline pretreatment of biomass is in general highly effective for hemicellulose removal, but the specific results depend on both the type of biomass and catalyst concentration. Another important effect of alkaline pretreatment methods is lignin solubilization. In this particular case, lignin content of collected WIS increased from 13% in the non-pretreated substrate to 23–26% (Table 3). However, such an increase can also be attributed to the solubilization of extracts, as commented previously for carbohydrate content. A similar trend was observed by Doménech et al. [38], who reported an increase of lignin content in pretreated olive stone from 33.8% in the raw material to 39.0% and 41.9% after extrusion at 100 °C with 5 and 10 g NaOH/100 g of olive stone, respectively. An increase in lignin content from 17.8% to 26.4–27.3% was also observed by Negro and co-workers [31] after subjecting olive tree pruning biomass to alkaline extrusion with 5 and 10% g NaOH/g dry matter ratio and a barrel temperature of 70–110 °C. In contrast, Coimbra et al. [39] reported a decrease in lignin content from 19.8% to 15.4% by subjecting wheat straw to extrusion at 70 °C and 6 g NaOH/100 g of substrate. Duque et al. [25] also reported a decrease in lignin content after extrusion pretreatment of barley straw from 15.2% to 14.5–15.6%, using 100 °C as barrel temperature and 5 and 7% NaOH/barley straw ratio on DW basis. Lignin content also ranged from 11.2 to 20.8% after extrusion pretreatment of *Mischantus* biomass with 0.3–0.9 M NaOH and 50–100 °C temperature range, compared to the 25.1% of lignin content measured in untreated biomass [29].

The liquid fractions resulting after the extrusion process were also collected and analyzed in terms of sugar content. Table 4 lists the concentration of the main sugar components identified in these liquid fractions.

About 4–5% of the initial sugars present in the raw material were found in the liquid fractions collected during extrusion pretreatment. Sugars were present mostly in oligomeric form, and a post-hydrolysis step was performed to determine the total sugars. According to the sugar profile in the collected soluble fractions, it seems that the concentration of catalyst did not influence the sugar release. In contrast, the number of runs (i.e., the increase in pretreatment time) had a positive effect on the production of sugars (*p* < 0.05). Glucose was the main sugar in the liquid fraction, and some nonstructural carbohydrates such as sucrose and fructose were also identified. Most of the sugars found in these liquid fractions mainly derived from the carbohydrates present in the aqueous extracts of the raw material and the solubilization of hemicelluloses. Acetic acid was also detected in the liquid fraction in the range of 0.3–0.9 g/100 g of VTPW. The production of acetic acid can be explained by the action of NaOH on the acetyl groups present in hemicelluloses. However, it is worth mentioning that the hydrolysis of acetyl groups from the hemicellulosic sugars was not complete, as some proportion of acetyl groups still remained in the WIS fraction (Table 3).

In terms of carbohydrate recovery, glucan recovery varied from 81–97% and 70–93% in the one-run and three-run assays, respectively. On the other hand, hemicellulose recoveries were 85–99% in the one-run test and 57–90% in the three-run assays. Overall, lower sugar recovery yields were observed when using higher concentrations of NaOH.

### 3.3. Enzymatic Hydrolysis of Pretreated Vegetal Tomato Plant Waste

**Figure 2:**
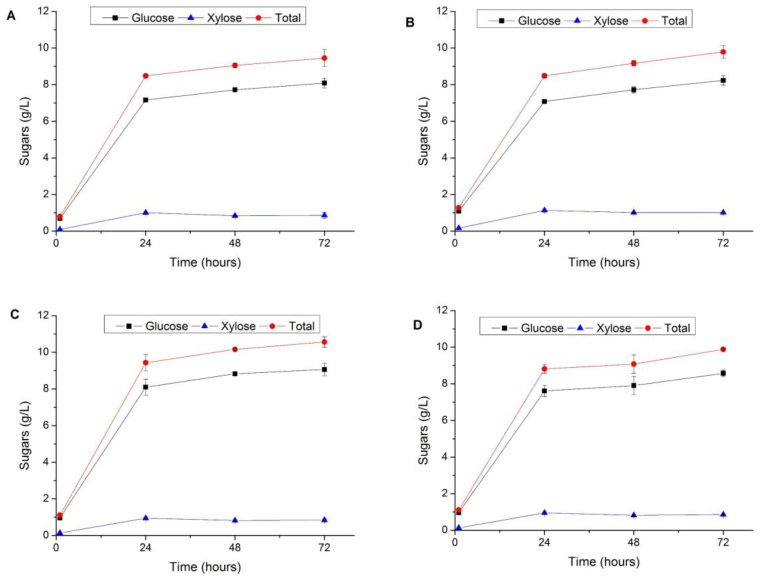
Sugar production by enzymatic hydrolysis of the water-insoluble solid (WIS) fractions collected after extrusion of vegetal tomato plant waste (VTPW). Enzymatic hydrolysis conditions: 50 °C, 5% (*w/v*) substrate loading, 15 FPU Cellic CTec2/g DW. (**A**) 6% NaOH (*w/w*), one-run: (**B**) 6% NaOH (*w/w*), three-run; (**C**) 12% NaOH (*w/w*), one-run; 12% NaOH (*w/w*), three-run. At the same time point, no statistically significant differences betwen any pair of means of sugar production were found for different extrusion treatment (**A**–**D**) at the 95.0% confidence level (one-way ANOVA with Bonferroni’s post-test was used for multiple comparison test, *n* = 3).

The main objective of pretreatment is to alter the structure of the fibers to increase the accessibility of enzymes to structural glucan and hemicellulose carbohydrates. After extrusion pretreatment, collected WIS fractions were subjected to an enzymatic hydrolysis test at 5% DW (*w/w*) substrate loadings to assess the sugar released and determine the hydrolysability potential of this pretreated substrate (Figure 2). Although the sugar concentration was quite similar in all pretreatment conditions, the increase in the pretreatment process time and catalyst concentration resulted in higher enzymatic hydrolysis yields. By using 6% (*w/w*) NaOH, EH_G_ increased from 65.2 in one-run to 73.7% in three-run respectively, whereas the use of 12% (*w/w*) NaOH increased EH_G_ yields from 76.2 to 81.3%. Similar saccharification yields to those observed for WIS-EVTPW in this study were obtained during the extrusion pretreatment of olive stone (15% NaOH/g DW of substrate and 125 °C) that yielded 7.6 and 6.2 g/L of glucose and xylose, corresponding to 60.8% and 61.2% of the total glucan and xylan, respectively [38].

Enzymatic hydrolysis tests at 10% (*w/v*) substrate loadings were also studied with the aim of increasing the resulting sugar concentration (Table 5). For these assays, in addition to estimate saccharification yields based on pretreated solid biomass (WIS), overall sugar yields were calculated based on the raw material (VTPW) to determine the pretreatment efficiency on the saccharification process. Considering this parameter, higher overall sugars yields were obtained for the 1-run assays independently of the NaOH concentration (*p* < 0.05), reaching 9.8–9.9 g glucose/100 g VTPW and 0.9–1 g xylose/100 g VTPW. These yields are equivalent to about 70% and 50% of the potential glucose and xylose present in the raw material, respectively. However, based on pretreated biomass, the 3-run assays with 12% (*w/w*) NaOH resulted in higher glucose (79.4%) and xylose (56.7%) yields.

Lower enzymatic hydrolysis yields were found for the xylan component. Although the Cellic CTec2 used for these tests also contained some xylan-related activities (e.g., xylanases and β-xylosidase), it is mainly considered a cellulolytic preparation. Supplementation of Cellic CTec2 with specific xylan-degrading preparations (such as Cellic HTec2) might contribute to improving saccharification performance of the pretreated WIS by increasing xylose release, therefore reaching higher yields.

### 3.4. Chemical Composition of Culled Tomato

Before subjecting culled tomato to the corresponding fractionation process, this residue was analyzed in order to identify its major components (Table 6). It is important to highlight that despite other macromolecular components, CT had a moisture content as high as 94.1%. Therefore, water could be identified as the main component of this residue. Apart from water, CT showed 69.1% of extractable compounds, of which 95.4% corresponded to aqueous extracts. This water-base extract contained up to 42.7/100 g DW of CT of sugars, mainly identified in the monomeric form. Fructose and glucose showed the highest concentrations, with 23.7% and 17.6%, respectively (Table 7).

Structural carbohydrates were the second most important component in CT, with a total content of 12.5% (Table 6). Among them, glucan was the principal constituent with 8.0 g/100 g DW of CT. The acid-insoluble solids and nitrogen content in CT was 9.3% and 1.5%, respectively. By considering a protein/nitrogen factor of 6.25, the protein content in CT could be estimated at 9.3%. This is an important value to be taken into account for the valorization of this residue, for instance, during some microbial oil production processes where nitrogen content should be low to trigger lipid accumulation [40].

Overall, CT showed a total of 55.2 g of carbohydrates per 100 g DW, which could be subsequently converted by means of biological processes under a wide range of fermentation-based strategies. Furthermore, it is worth mentioning that around 80% of these potential sugars are found in the aqueous fraction, which therefore make them easily available for their downstream processing and might facilitate the scale up of the process at the industrial level.

### 3.5. Fractionation of Culled Tomato and Enzymatic Hydrolysis

CT was sequentially subjected to crushing and centrifugation steps, allowing fractionation and collection of the corresponding LF-CT and SF-CT fractions. Table 7 and Table 8 list the complete characterization of both LF-CT and SF-CT. In total, 1 kg of CT (having 59.2 g of total solids) yielded 39.4 g of sugars per 100 g DW of CT in the LF-CT without any further treatment. As commented previously, this represents a great advantage, since most sugars can be extracted without the need of subjecting CT to harsh conditions, as is the case for lignocellulosic-based materials (e.g., VTPW). On the other hand, SF-CT mainly contains structural polysaccharides and therefore requires an enzymatic hydrolysis process with cellulases/hemicellulases to release the corresponding sugars monomers.

**Figure 3:**
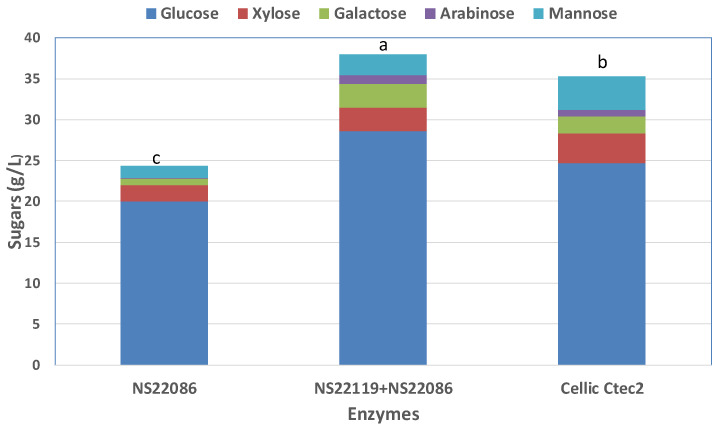
Sugar production from the solid fraction of culled tomato (SF-CT) after 48 h of enzymatic hydrolysis with different enzyme blends. Description about enzyme activities can be found in the Methods section. ANOVA analysis was performed for the total sugar (glucose + xylose + galactose + arabinose + mannose), and statistical differences at the 95% level are represented by different letters (a–c). One-way ANOVA with Bonferroni’s post-test was used for multiple comparison test, *n* = 3.

Enzymatic hydrolysis of SF-CT was performed at 10% (*w/w*) substrate concentration with different enzyme blends to identify the main activities providing effective saccharification of this feedstock (Figure 3). The commercial preparation Cellic CTec 2 was used as the base-case study, while the enzyme complex NS22086 alone or in combination with NS22119 was tested as enzyme mixtures with different carbohydrolase activities (e.g., arabinases, β-glucanases, cellulases, pectinases and xylanases). Supplementation of NS22086 with NS22119 is recommended by the supplier for substrates with a high pectin content (data from Provider).

The highest sugar released during enzymatic hydrolysis of SF-CT was reached with the combination of the enzyme complex NS22086 and NS22119. Compared to the base-case study, this enzyme blend increased the total sugar concentration by about 10%, from 35.2 g/L to 38.0 g/L. Independently of the enzyme mixture used, glucose was the main sugar monomer, reaching a maximum concentration of 28.0 g/L with the NS22086 + NS22119 blend, which corresponded to a glucose yield of 92%.

In brief, after the fractionation step of CT and enzymatic hydrolysis of SF-CT, 9.1 g glucose/100 g DW of CT residues could be obtained.

### 3.6. Sugars Production from the Organic Residues Derivied from Tomato Greenhouse Crops

**Figure 4:**
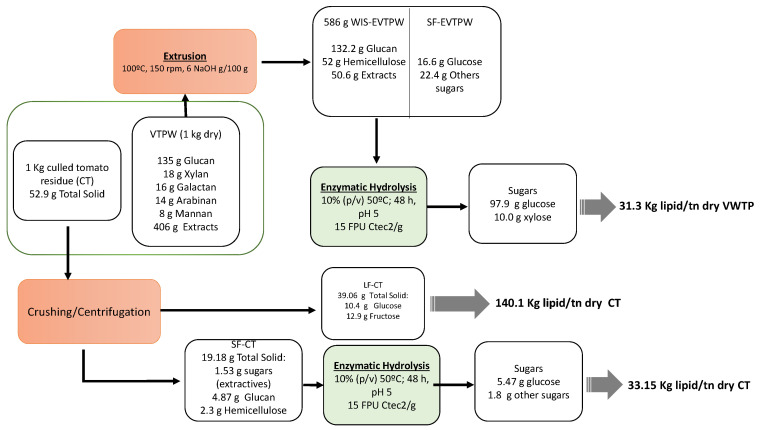
Sugar mass balance from organic residues from tomato greenhouse crops.

The aim of this work was to study the sugar production from VTPW and CT to evaluate the suitability of these feedstocks as commodities for the production of industrially relevant bio-based products, such as microbial oil. The complete sugar mass balance from the organic residues derived from tomato greenhouse crops is depicted in Figure 4. Considering sugar production from VTPW and CT residues and the maximum stoichiometric sugar-to-lipid conversion yields for oleaginous yeasts [41], 31.3 and 173.2 kg of lipids/ton DW of either VTPW or CT may be obtained from these residues. These yields have been estimated considering glucose and fructose sugars only. However, higher yields might be obtained when using microbial strains capable of utilizing all the sugars monomers released from these sources.

VTPW: vegetal tomato plant waste; WIS-EVTPW: Water insoluble solid from extrudate vegetal tomato plant waste; SF-EVTP: soluble fraction from extrudate vegetal tomato plant waste; CT: Culled tomato residue; LF-CT: liquid fraction from culled tomato residue; SF-CT: solid fraction from culled tomato residue.

## 4. Conclusions

Tomato is the major vegetable crop in Europe, with Almería as one of the main tomato production areas. During tomato cultivation and harvesting, a considerable amount of organic waste is produced, including VTPW and CT. Even considering a biorefinery based on a single industry such as the tomato crop, the different nature of the vegetal residues generated asks for an individualized approach to each of them. The complete characterization of the potential feedstocks is a first step that supports the choice of the best fractionation technique that would make the most of each substrate. In this regard, VTPW, a lignocellulosic biomass with a considerable extractable fraction, was submitted to alkali-catalyzed extrusion pretreatment as a means to concentrate carbohydrates and enhance their enzymatic digestibility. This pretreatment resulted in high sugar recovery (>80% for 1 run and 6% *w/w* NaOH) and improved the enzymatic accessibility of the substrate, resulting in the conversion of about 70% and 50% of the potential glucose and xylose in the raw material. On the other hand, CT analysis revealed that 43% of the biomass dry weight were free soluble sugars that could be easily obtained in the juice after a simple crushing and centrifugation process. Furthermore, the resulting solid fractions were subsequently subjected to enzymatic hydrolysis in order to recover the sugars from structural carbohydrates and add them up to the process. Having as example the conversion of sugars into microbial oil, about 175 kg of lipids/ton DW of CT and 31.3 kg of lipids/ton DW of VTPW may be obtained from these residues.

## Figures and Tables

**Table 1:** VTPW from greenhouse crop composition. Data expressed as percentage (*w/w*) on a dry weight (DW) basis.

Component	% (*w/w*)
Extracts	40.6 ± 0.4
Aqueous extract	35.0 ± 0.3
Organic solvent- extract	5.6 ± 0.3
Glucan	13.5 ± 0.1
Hemicelluloses	5.6 ± 0.1
Xylan	1.8 ± 0.1
Galactan	1.6 ± 0.1
Arabinan	1.4 ± 0.0
Mannan	0.8 ± 0.0
Acetyl groups	1.1 ± 0.0
Acid-insoluble lignin	9.4 ± 1.1
Acid-soluble lignin	3.6 ± 0.3
Whole Ash	17.5 ± 0.7

**Table 2:** Water insoluble content in extrudates and mass yield after extrusion pretreatment. Data expressed as percentage (*w/w*) on a DW basis. Values followed by different letter in the same row are significantly different at the 95% level.

Parameter	One-Run		Three-Run	
	6% NaOH (*w/w*)	12% NaOH (*w/w*)	6% NaOH (*w/w*)	12% NaOH (*w/w*)
g WIS/100 g extrudate	56.6 ^a,b^	49.5 ^b,c^	56.9 ^a^	46.8 ^c^
Mass yields (%)	58.7 ^a^	54.4 ^b^	58.3 ^a^	51.1 ^b^

**Table 3:** Chemical composition of the water-insoluble solid (WIS) fraction collected after extrusion of the vegetal tomato plant waste (VTPW). Data expressed as percentage (*w/w*) on a DW basis. Values followed by different letter in the same row are significantly different at the 95% level.

Component	Extrusion Conditions			
	One-Run		Three-Run	
	6% NaOH (*w/w*)	12% NaOH (*w/w*)	6% NaOH (*w/w*)	12% NaOH (*w/w*)
Extracts	8.6 ± 0.5	12.8 ± 0.7	6.8 ± 0.4	7.1 ± 0.5
Glucan	22.5 ± 0.8 ^a^	20.3 ± 0.9 ^ab^	21.6 ± 0.2 ^ab^	19.2 ± 0.7 ^b^
Xylan	3.6 ± 0.2 ^b^	2.8 ± 0.1 ^a^	3.9 ± 0.3 ^b^	2.5 ± 0.1 ^a^
Galactan	3.4 ± 0.2 ^b^	3.3 ± 0.1 ^b^	3.2 ± 0.1 ^b^	2.5 ± 0.1 ^a^
Arabinan	1.8 ± 0.1 ^ab^	1.9 ± 0.1 ^a^	1.7 ± 0.0 ^b^	1.3 ± 0.1 ^c^
Mannan	1.1 ± 0.1 ^b^	0.9 ± 0.0 ^b^	1.1 ± 0.0 ^a^	0.7 ± 0.0 ^c^
Acetyl-groups	0.6 ± 0.0	0.4 ± 0.0	0.6 ± 0.0	0.4 ± 0.0
Lignin	23.0 ± 0.4 ^b^	26.0 ± 0.7 ^ab^	23.1 ± 0.6 ^ab^	23.1 ± 0.3 ^a^
Ash	14.8 ± 0.1 ^b^	16.9 ± 0.1 ^a^	17.1 ± 0.1 ^a^	17.0 ± 0.2 ^a^

**Table 4:** Sugar composition for the soluble fractions collected during each extrusion pretreatment. Data expressed as g/100 g of extrudate.

Component	Extrusion Conditions			
	One-Run		Three-Run	
	6% NaOH (*w/w*)	12% NaOH (*w/w*)	6% NaOH (*w/w*)	12% NaOH (*w/w*)
Sucrose	0.5	0.4	0.6	0.4
Glucose	1.7	1.8	2.4	2.7
Xylose	0.1	0.2	0.1	0.1
Galactose	0.6	1.1	0.8	1.1
Arabinose	0.3	0.3	0.2	0.2
Mannose	0.1	0.2	0.2	0.2
Fructose	0.7	0.1	0.7	0.3
Total sugars	3.9	4.0	4.9	5.0

**Table 5:** Enzymatic hydrolysis yield, glucose and xylose produced by 100 g DW of the water-insoluble solid (WIS) fraction collected after extrusion of vegetal tomato plant waste (VTPW), and overall glucose and xylose yield. Values followed by different letters in the same row are significantly different at the 95% level.

Extrusion Conditions		EH_g_ (%)	Glucose Yield (g/100 g WIS)	Overall Glucose Yield (g/100 g VTPW)	EH*_x_* (%)	Xylose Yield (g/100 g dry WIS)	Overall Xylose Yield (g/100 g VTPW)
6% (*w/w*) NaOH	One-run	67.3 ^b^	16.7 ^a^	9.8 ^a^	41.4 ^b^	1.7 ^a^	1.0 ^a^
	Three-run	71.8 ^ab^	16.0 ^a^	8.7 ^a^	57.5 ^a^	1.8 ^a^	1.0 ^a^
12% (*w/w*) NaOH	One-run	71.6 ^ab^	17.0 ^a^	9.9 ^a^	47.1 ^ab^	1.5 ^a^	0.9 ^a^
	Three-run	79.4 ^a^	16.8 ^a^	8.6 ^a^	56.7 ^a^	1.6 ^a^	0.8 ^a^

**Table 6:** Chemical composition of culled tomato residue. Data expressed as percentage (*w/w*) on a DW basis.

Component	% (*w/w*) DW
Total extract	69.1 ± 5.2
Aqueous extract	65.9 ± 4.5
Organic extract	3.3 ± 1.0
Structural carbohydrates	12.5 ± 0.1
Glucan	8.0 ± 0.1
Xylan	1.3 ± 0.1
Galactan	1.3 ± 0.0
Arabinan	0.6 ± 0.0
Mannan	1.3 ± 0.1
Acid Insoluble solids	9.3 ± 0.8
Ash	6.9 ± 0.1
Nitrogen	1.5 ± 0.1

**Table 7:** Sugars composition in aqueous extract in culled tomato residue. Data expressed as percentage (*w/w*) on a DW basis.

Sugars	% (*w/w*) DW	
	Monomers	Oligomers
Glucose	17.6 ± 3.0	0.1 ± 0.0
Xylose	0.3 ± 0.0	0.1± 0.0
Galactose	0.3 ± 0.1	0.9 ± 0.1
Arabinose	0.1 ± 0.1	0.6 ± 0.0
Mannose	0.5 ± 0.1	n.f.
Fructose	23.7 ± 2.0	n.f.
Sucrose	0.3 ± 0.0	

n.f., not found.

**Table 8:** Composition of the solid fraction (SF-CT) obtained after crushing and centrifugation of culled tomato residue (CT). Data expressed as percentage (*w/w*) on a DW basis.

Component	% (*w/w*) DW
Total extract	31.5 ± 0.4
Aqueous extract	20.7 ± 0.3
Sugars	8.3 ± 0.2
Inorganic compounds	3.3 ± 0.1
Organic extract	10.8 ± 0.2
Structural carbohydrates	37.1 ± 0.3
Glucan	25.4 ± 0.4
Xylan	3.3 ± 0.2
Galactan	2.9 ± 0.1
Arabinan	1.3 ± 0.1
Mannan	4.30 ± 0.1
Ash	4.6 ± 0.2
Nitrogen	2.5 ± 0.0

## Data Availability

All data generated or analyzed during this study are included in this published article.
